# Minimally invasive treatments for benign prostatic enlargement: systematic review of randomised controlled trials

**DOI:** 10.1136/bmj.a1662

**Published:** 2008-10-09

**Authors:** Tania Lourenco, Robert Pickard, Luke Vale, Adrian Grant, Cynthia Fraser, Graeme MacLennan, James N’Dow

**Affiliations:** 1 Health Services Research Unit, Institute of Applied Health Sciences, University of Aberdeen; 2 Department of Urology, School of Surgical and Reproductive Sciences, Newcastle University, Newcastle upon Tyne; 3 Health Economics Research Unit, Institute of Applied Health Sciences, University of Aberdeen; 4 Academic Urology Unit, Institute of Applied Health Sciences, University of Aberdeen, Aberdeen AB25 2ZD

## Abstract

**Objective** To compare the effectiveness and risk profile of minimally invasive interventions against the current standard of transurethral resection of the prostate.

**Design** Systematic review and meta-analysis of randomised controlled trials.

**Data sources** Electronic and paper records up to March 2006.

**Review methods** We searched for all relevant randomised controlled trials. Two reviewers independently extracted data and assessed quality. Meta-analyses of prespecified outcomes were performed with fixed and random effects models and reported using relative risks or weighted mean difference.

**Results** 3794 abstracts were identified; 22 randomised controlled trials met the inclusion criteria. These provided data on 2434 participants. The studies evaluated were of moderate to poor quality with small sample sizes. Minimally invasive interventions were less effective than transurethral resection of the prostate in terms of improvement in symptom scores and increase in urine flow rate, with most comparisons showing significance despite wide confidence intervals. Rates of reoperation were significantly higher for minimally invasive treatments. The risk profile of minimally invasive interventions was better than that of transurethral resection, with fewer adverse events. The results, however, showed significant heterogeneity.

**Conclusion** Which minimally invasive intervention is the most promising remains unclear. Their place in the management of benign prostate enlargement will continue to remain controversial until well designed and well reported randomised controlled trials following CONSORT guidelines prove they are superior and more cost effective than drug treatment, or that strategies of sequential surgical treatments are preferred by patients and are more cost effective than the more invasive but more effective tissue ablative interventions such as transurethral resection.

## Introduction

Benign prostatic enlargement is the commonest cause of lower urinary tract symptoms such as frequency of urination and slow stream, which are highly prevalent among older men. Endoscopic removal of the inner part of the gland by transurethral resection of the prostate has long been considered as the most effective treatment, with about 20 000 procedures carried out in 2007 in the United Kingdom. This procedure has known disadvantages, including blood loss and physiological stress, which affect recovery in some men, together with the need for high levels of technical skill and a three to five day stay in hospital.^1^ These factors have encouraged the development of alternative treatments, including drugs, minimally invasive surgical techniques, and different forms of endoscopic prostatectomy, with the intention of achieving the same degree of improvement of symptoms but with less morbidity and lower cost. Minimally invasive techniques aim to reduce prostate volume by delayed tissue necrosis using relatively low levels of thermal energy and thus reducing the risk of morbidity related to treatment.

We carried out a systematic review to summarise evidence of benefit and risk for seven promising minimally invasive technologies compared with transurethral resection using data from randomised controlled trials.

## Methods

### Search strategy

To identify published and unpublished reports of relevant randomised controlled trials we carried out highly sensitive electronic searches of relevant databases and recent conference proceedings (Medline (1966-September, week 3, 2006), Embase (1980-2006, week 38), Medline In-Process (27 September 2006), Biosis (1985-22 September 2006), Science Citation Index (1981-23 September 2006), ISI Proceedings (1990-18 March 2006), Cochrane Controlled Trials Register and Cochrane Database of Systematic Reviews (Cochrane Library, Issue 1, 2006), Database of Abstracts of Reviews of Effectiveness (March 2006), HTA Database (March 2006), National Research Register (Issue 1, 2006), Clinical Trials (March 2006), Current Controlled Trials (March 2006), and proceedings of the European Association of Urology, American Urological Association and the British Association of Urological Surgeons). We defined minimally invasive treatments as those that reduce prostate volume by delayed tissue necrosis using low energy heating devices. Searches were not restricted by year of publication or language and included abstracts from conference proceedings. Reference lists of all included studies were scanned to identify additional potentially relevant studies. Full details of the search strategies used are available from the authors on request. Two reviewers (from TL and Angela Coutts or Susan Wong or Ghulam Nabi) independently screened the titles and abstracts of identified papers, and full text copies of all potentially relevant studies were obtained.

### Selection and study characteristics

We included randomised controlled trials if they compared minimally invasive interventions for benign prostatic enlargement with transurethral resection of the prostate. We excluded trials reporting on men without a clinical diagnosis of benign prostatic enlargement and comparisons against conservative management. The table shows the interventions considered (table A on bmj.com provides full descriptions of the treatments).[Table tbl1] The primary outcome measure was change in symptom score 12 months after surgery, measured by the international prostate symptom score or the American Urological Association symptom index—these were considered equivalent and we therefore combined trials using these instruments. The international prostate symptom score/American Urological Association symptom questionnaire asks men to rate four voiding symptoms (poor stream, intermittent flow, straining, incomplete emptying) and three storage symptoms (frequency, nocturia, urgency) on a scale from 0 (not present) to 5 (severe) to give a total score ranging from 0 to 35, with severity of symptoms defined as mild (0-7), moderate (8-19), or severe (20-35).

Secondary outcomes were improvement in quality of life and increase in peak urine flow rate (for effectiveness); blood transfusion, urinary incontinence, urinary retention, urethral stricture, urinary tract infection, retrograde (loss of) ejaculation, erectile dysfunction (for morbidity); and duration of operation, length of hospital stay, and need for reoperation (for descriptors of care). We considered all reports of prespecified complications regardless of their timing. As separate reporting of stenosis of the bladder neck and urethral stricture was inconsistent we combined these complications.

**Table tbl1:** Details of 22 included studies (22 trials with 24 relevant comparisons) and summary of baseline characteristics (means or medians)

Study	Comparators	No of participants	Age (years)	Symptom score*	Qmax ml/s	Residual volume (ml)	Prostate size (ml)
Ahmed 1997^w1^	TUMT *v* TURP	30/30	69/69	18.5/18.4	10.1/9.5	94/109	37/46
Dahlstrand 1993^w2^	TUMT *v* TURP	39/40	68/70	11.2†/13.3†	8.0/7.9	105/116	33/37
Dahlstrand 1995^w3^	TUMT *v* TURP	37/32	67/70	12.1†/13.6†	8.6/8.6	194/1104	43‡/45‡
D’Ancona 1998^w4^	TUMT *v* TURP	31/21	69/69	18.3/16.7	9.3/9.3	49/91	43/45
De la Rosette 2003^w5^	TUMT *v* TURP	78/66	67/66	20.0/20.0	9.2/8.0	65/91	51/52
Wagrell 2002^w6^	TUMT *v* TURP	99/46	67/69	21.0/20.4	7.6/7.8	106/94	49/53
Cimentepe 2003^w7^	TUNA *v* TURP	26/33	60/63	22.9/24.1	9.8/9.2	67/76	46/49
Hill 2004^w8^	TUNA *v* TURP	65/56	66/66	23.9/24.1	8.8/8.8	92/83	36/36
Hindley 2001^w9^	TUNA *v* TURP	25/25	66§/71§	22§/20§	8.5/9.0	55/74	NR/NR
Kim 2006^w10^	TUNA *v* TURP	110/110	66/67	20.8/24.0	7.0/11.9	257/187	41/44
Kim 2006^w10^	TEAP *v* TURP	94/110	66/67	19.5/24.0	7.2/11.9	126/187	36/44
Aliaga 1998^w11^	Laser coagulation *v* TURP	18/21	NR	25.5/24.2	7.0/8.3	77/89	20-60 g/20-60 g
Chacko 2001^w12 ^(Clasp study)	Laser coagulation *v* TURP	74/74	74/73	17.6/19.4	NR	NR	NR
Costello 1995^w13^	Laser coagulation *v* TURP	34/37	68/68	NR	8.76/9.48	NR	30/34
Cowles 1995^w14^	Laser coagulation *v* TURP	56/59	65/67	18.7/20.8	8.9/9.5	163/207	42/39
Donovan 2000^w15^ (Clasp study)	Laser coagulation *v* TURP	117/117	67/66	19.1/19.2	10.4/10.3	124/104	41/38
Gujral 2000^w16 ^(Clasp study)	Laser coagulation TURP	38/44	70/70	20.9/19.5	11.2/8.5	438/545	41§/50§
Kabalin 1995^w17^	Laser coagulation *v* TURP	13/12	65/69	20.9/18.8	8.5/9.0	236/291	24‡/17‡
Kim 2006^w10^	Laser coagulation *v* TURP	89/110	69/67	21.1/24.0	8.6/11.9	219/187	43/44
Kursh 2003^w18^	Laser coagulation *v* TURP	37/35	68/69	24.0§/23.0§	9.2§/9.1§	81§/87§	41§/40§
Liedberg 2003^w19^	Laser coagulation TURP	20/11	NR/NR	19§/17§	8§/8§	96§/117§	49§/47§
Mårtenson 1999^w20^	Laser coagulation *v* TURP	30/14	>45/>45	21.7/21.6	7.3/9.3	116/88	46/50
McAllister 2000^w21^	Laser coagulation *v* TURP	76/75	68/68	18.1/18.2	9.6/10.0	113/120.7	NR
Suvakovic 1996^w22^	Laser coagulation *v* TURP	10/10	67/66	15.7/18.8	10.5/11.1	47/162	24 g/22 g

NR=not reported; TUMT=transurethral microwave thermotherapy; TUNA=transurethral needle ablation; TEAP=transurethral ethanol ablation of prostate.

*International prostate symptom score/American Urological Association symptom index.

†Madsen score.

‡Prostate length (mm).

§Median.

### Validity assessment and data abstraction

Two reviewers (as above), independently assessed the quality of methods of the full text studies using an assessment tool drawing on the schema suggested by the National Health Service Centre for Reviews and Dissemination,^2^ Verhagen et al,^3^ Downs and Black,^4^ and the generic appraisal tool for epidemiology. Judgments on quality were based on the qualitative assessment of the number and type of criteria met by individual studies.

The two reviewers recorded details of methods, interventions, participants’ characteristics, and outcomes on a data extraction form. Any differences that could not be resolved through discussion were decided by an arbiter. For trials with multiple publications we included only the most complete report for each outcome.

### Quantitative data synthesis

For meta-analysis we combined dichotomous outcome data using the Mantel-Haenszel relative risk method. For continuous outcomes we used inverse variance weighted mean differences and 95% confidence intervals. We intended to report results using a fixed effects model throughout, but we had to use a random effects model for symptom score and peak urine flow rate because of statistical heterogeneity, explored by χ^2^ tests and I^2^ statistics. We used the standard Cochrane software RevMan 4.2.8 for meta-analyses.

## Results

The initial search generated 3794 reports, of which 621 were selected for full assessment (see fig A on bmj.com). Fifty four reports describing 22 trials met the eligibility criteria (table).[Table tbl1]

We classed the 22 full text randomised controlled trials^w1-w22^ as being moderate or poor quality (see table B on bmj.com). Only eight studies (36%)^w6 w11 w12 w14-w16 w18 w21^ explicitly stated that an intention to treat analysis was performed and in seven of these, this was compromised by failure to include the total number of participants in each arm at subsequent follow-up assessments.^w6 w12 w14-w16 w18 w21^ A further trial was compromised by allowing substitution of patients who failed to complete their assigned treatment or did not attend for follow-up.^w1^ None of the trials provided information on whether the interventions were undertaken by someone experienced in performing the procedure, and some studies were unclear about how many patients were assessed at each follow-up. Trial setting and baseline characteristics of the patients, such as disease severity measured by international prostate symptom score, varied across the included trials. Types of laser and delivery mode of laser coagulation also varied (see table C on bmj.com). The eligible trials included 24 relevant comparisons involving 2434 participants. We could not assess differences in outcome between transurethral resection and high intensity focused ultrasound, water thermotherapy, and transurethral ethanol ablation of prostate because of the lack of data.

### Quantitative data synthesis

*Symptom scores*—Results from studies reporting change in symptom score from baseline to 12 months showed significant heterogeneity so we carried out random effect meta-analyses (fig 1). There was generally less improvement in symptom score for patients who underwent transurethral microwave treatment, transurethral needle ablation, and laser coagulation than transurethral resection of the prostate (table D on bmj.com): weighted mean difference 2.26, 95% confidence interval −0.38 to 4.91, P=0.09, after transurethral microwave treatment; mean difference 3.90, 1.27 to 6.53, P=0.004, after transurethral needle ablation; weighted mean difference 3.01, −1.06 to 7.07, P=0.15, after laser coagulation.

**Figure fig1:**
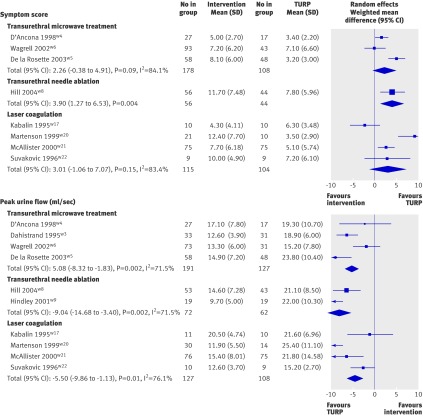
**Fig 1** Symptom scores (international prostate symptom score or American Urological Association symptom index) and peak urine flow rate at 12 months (TURP=transurethral resection of prostate)

*Peak urine flow rate*—The results for peak urine flow rate at 12 months were generally consistent with those for symptom scores (fig 1).[Fig fig1] Results of four trials that presented data that could not be included in the meta-analysis were consistent with these findings: weighted mean difference −5.08, −8.32 to −1.83, P=0.002, after transurethral microwave treatment; −9.04, −14.68 to −3.40 , P=0.002, after transurethral needle ablation; −5.50, −9.86 to −1.13, P=0.01, after laser coagulation.

*Quality of life*—Two,^w5 w6^ four,^w7-w10^ and six^w10 w12 w15 w16 w18 w20^ studies comparing transurethral resection with transurethral microwave treatment, transurethral needle ablation, and laser coagulation, respectively, reported on quality of life using various instruments. Scores after these interventions were generally poorer than after transurethral resection, but we could not undertake a formal meta-analysis (see table D on bmj.com).

*Blood transfusion*—Blood transfusion was less common after minimally invasive treatments than after transurethral resection (fig 2): relative risk 0.11, 95% confidence interval 0.01 to 1.98, P=0.13, after transurethral microwave treatment; 0.05, 0.01 to 0.32, P=0.002, after transurethral needle ablation; 0.11, 0.04 to 0.26, P<0.001, after laser coagulation). No patients undergoing transurethral microwave treatment or transurethral needle ablation required a blood transfusion.

**Figure fig2:**
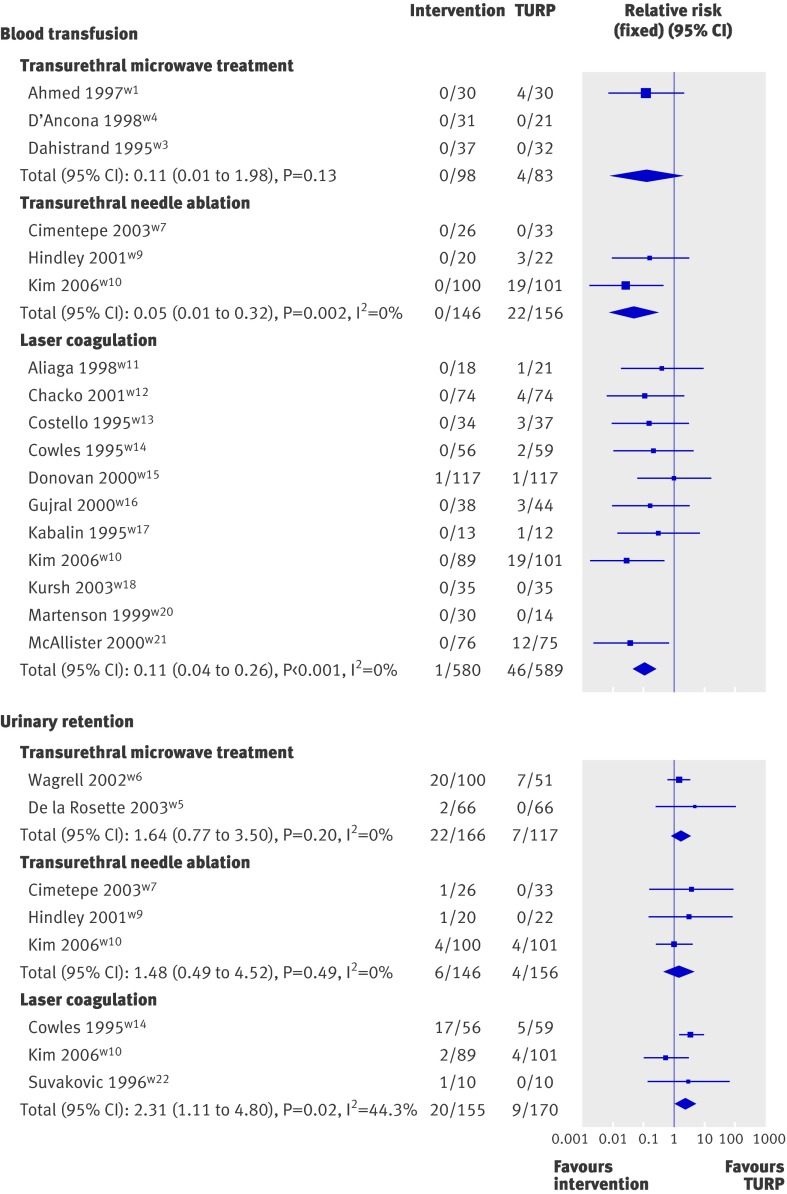
**Fig 2** Blood transfusion and urinary retention after interventions (TURP=transurethral resection of prostate)

*Urinary retention*—Laser coagulation was associated with a higher rate of urinary retention than transurethral resection (12.9% *v* 5.3%, relative risk 2.31, 1.11 to 4.80, P=0.02). No significant differences were seen between transurethral resection and transurethral microwave treatment or transurethral needle ablation (fig 2).[Fig fig2]

*Strictures*—Men undergoing a minimally invasive treatment had a lower risk of developing strictures (0.6% *v* 6.5%, relative risk 0.20, 0.05 to 0.75, P=0.02, after transurethral microwave treatment; 0.5% *v* 6.8%, 0.14, 0.03 to 0.62, P=0.009, after transurethral needle ablation; 0.9% *v* 8.6%, 0.18, 0.06 to 0.56, P=0.003, after laser coagulation) (see fig B on bmj.com).

*Incontinence*—The risk of postoperative incontinence was lower after transurethral needle ablation and laser coagulation than after transurethral resection (0.9% *v* 8.0%, relative risk 0.16, 0.05 to 0.51, P=0.002; 0% *v* 3.9%, 0.16, 0.04 to 0.71, P=0.02, respectively), while there was no significant difference after transurethral microwave treatment (4.9% *v* 8.3%, 0.61, 0.30 to 1.26, P=0.18) (see fig B on bmj.com).

*Urinary tract infection*—There was no significant difference in rates of urinary tract infection after transurethral microwave treatment and transurethral needle ablation compared with transurethral resection (6.7% *v* 7.5%; 1.05, 0.53 to 2.08, P=0.90, after transurethral microwave treatment; 10.3% *v* 7.0%; 1.42, 0.69 to 2.91, P=0.34, after transurethral needle ablation). The rate of postoperative urinary tract infection, however, was higher in patients after laser coagulation (14.8% *v* 6.9%; 1.84, 1.22 to 2.79, P=0.004) (see fig B on bmj.com).

*Sexual dysfunction*—Men undergoing a minimally invasive treatment were less likely to experience loss of ejaculation compared with those having transurethral resection. Also, after transurethral needle ablation or laser coagulation sexually active men had a lower risk of experiencing erectile dysfunction, but the difference between transurethral microwave treatment and transurethral resection was not significant (fig 3).

**Figure fig3:**
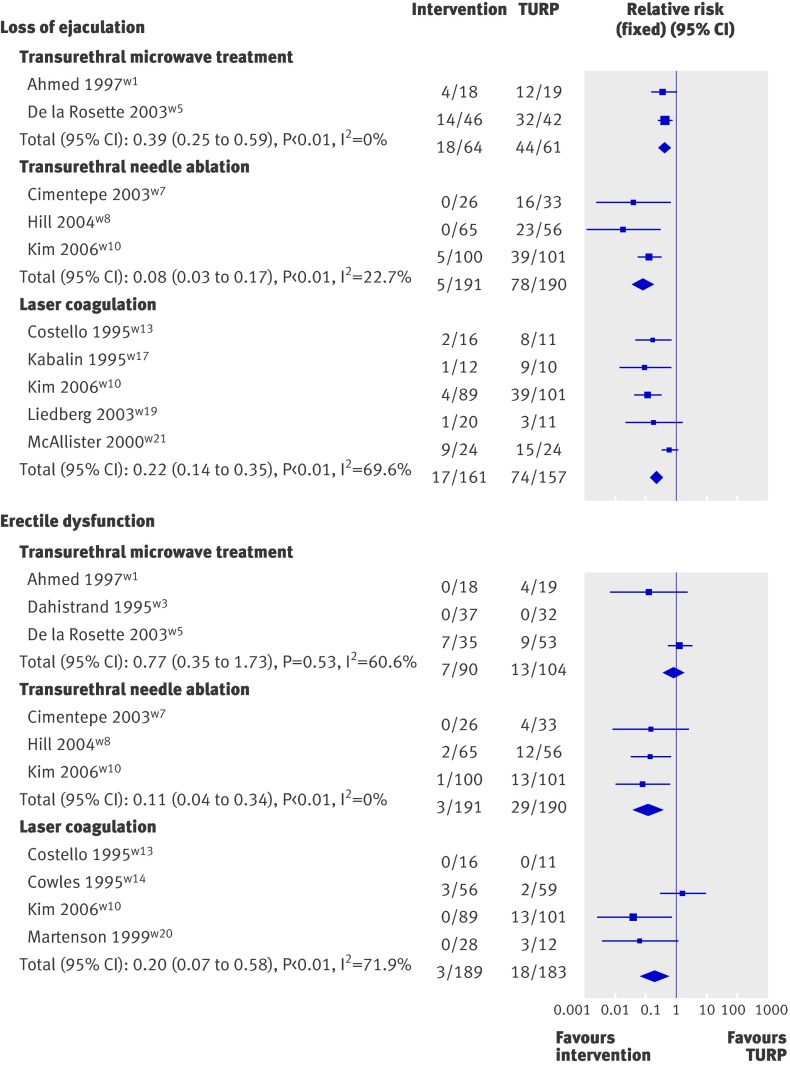
**Fig 3** Sexual function after interventions (TURP=transurethral resection of prostate)

*Descriptors of care*—Compared with transurethral resection, transurethral needle ablation procedures were longer and laser coagulation procedures shorter (fig 4). We had insufficient data to assess differences in duration of operation between transurethral microwave treatment and transurethral resection. Hospital stay was on average one day shorter for laser coagulation than for transurethral resection (95% confidence interval −1.68 to −0.98 days, P<0.001) (fig 5).[Fig fig5] This was consistent with results from other trials with data unsuitable for meta-analysis. Concerning transurethral needle ablation, two studies favoured transurethral resection of the prostate^ w7 w9^ and one favoured transurethral needle ablation.^w10^ Patients undergoing transurethral microwave treatment were generally treated as day cases and therefore hospital stay was clearly longer for transurethral resection. The need for reoperation was more common after a minimally invasive procedure (10.2% *v* 4.8%, 2.01, 0.96 to 4.18, P=0.06, after transurethral microwave treatment; 6.2% *v* 0.5%, 6.89, 1.58 to 29.95, P=0.01, after transurethral needle ablation; 7.9% *v* 2.0%, 3.21, 1.65 to 6.24, P<0.001, after laser coagulation) (fig 6).[Fig fig6]

**Figure fig4:**
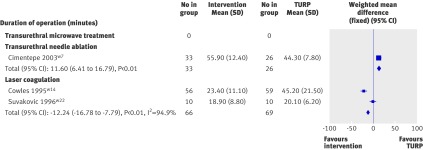
**Fig 4** Duration of operation (TURP=transurethral resection of prostate)

**Figure fig5:**
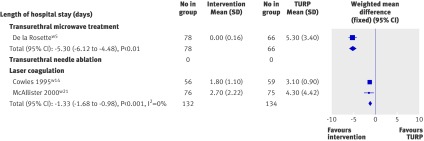
**Fig 5** Length of hospital stay after intervention (TURP=transurethral resection of prostate)

**Figure fig6:**
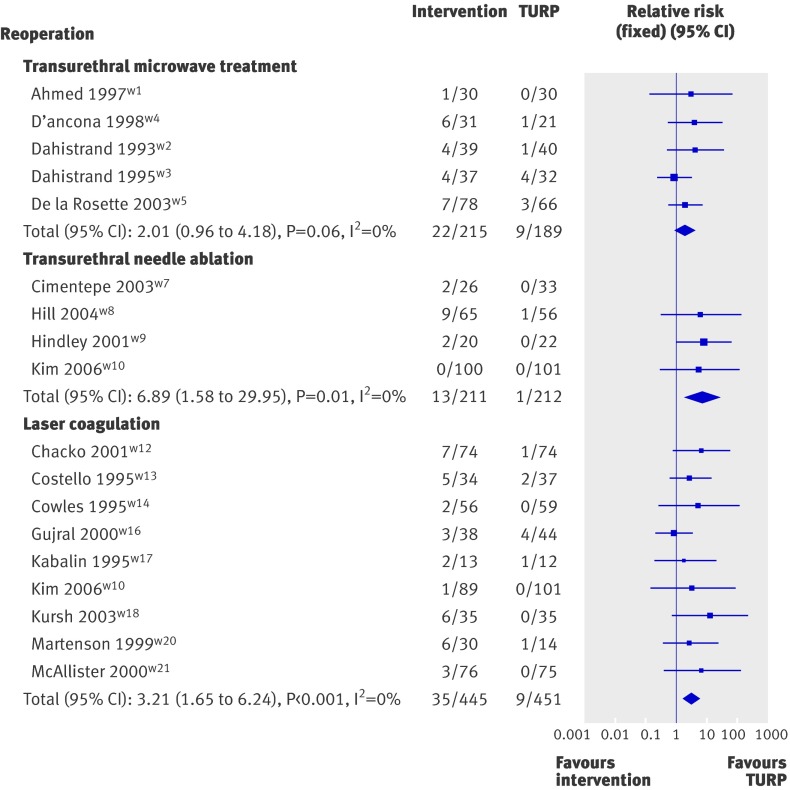
**Fig 6** Number of patients who underwent reoperation (TURP=transurethral resection of prostate)

## Discussion

### Principle findings

A single minimally invasive treatment of benign prostatic enlargement with transurethral microwave treatment, transurethral needle ablation, or laser coagulation results in improvement of symptoms at 12 months, but this is markedly inferior to that seen after transurethral resection of the prostate and in some trials is associated with significantly less improvement in quality of life. In our systematic review this difference in subjective treatment response was mirrored by the much smaller increase in peak urinary flow rate after a minimally invasive treatment, signifying reduced urodynamic benefit. The risk profile of minimally invasive interventions was generally superior to transurethral resection, with fewer adverse events in terms of the need for a blood transfusion, development of strictures, and urinary incontinence, but the risk of reoperation within the relatively short follow-up periods reported was between two and seven times higher than for transurethral resection. Overall, our findings do not support a change in surgical treatment of benign prostatic enlargement away from the current standard of transurethral resection. On an individual basis, however, personal preference will influence choice of procedure and some patients might trade-off the lower efficacy and higher risk of reoperation for the decreased morbidity seen with minimally invasive treatment options.

### Meaning of the study (possible mechanism and implications for policy and practice)

Although improvements in symptoms after minimally invasive treatments was inferior to transurethral resection of the prostate, it still represents a considerable treatment effect given that a reduction of more than 3 points in the international prostate symptom score is of noticeable benefit for men with benign prostatic enlargement.^5^ It might be better to use these techniques earlier in the treatment algorithm, possibly as alternatives to long term drug treatment with α adrenergic blockers or 5-α reductase inhibitors. One randomised controlled trial showed superior efficacy in terms of symptom scores, peak urine flow rate, and quality of life with single transurethral microwave treatment at six months after treatment compared with terazocin, an α adrenergic antagonist.^6^ The closer equivalence with drug treatment is also indicated by the substantially lower improvement in peak flow rate compared with transurethral resection, which is likely to reflect the smaller quantity of prostate tissue ablated. The potential benefits of this treatment strategy, however, require further evaluation given the greater risk of potentially serious adverse events such as infection and urinary retention and the need for more focused selection of patients compared with drug treatment.

Minimally invasive treatments for benign prostatic enlargement were originally conceived as an attempt to offer equivalent efficacy to transurethral resection without the burden and risk of operative morbidity. They have tended to be introduced in a haphazard and uncontrolled manner and have yet to find their place as approved treatments in healthcare systems such as the UK NHS. The main attraction to men considering such alternative treatments is the lower risk of adverse effects and shorter recovery time. Our meta-analysis showed that transurethral microwave treatment and transurethral needle ablation are less likely to lead to major blood loss, which is of undoubted benefit to men with major comorbidities. There was also evidence of lower risk of sexual side effects such as ejaculatory disturbance and erectile dysfunction, which presumably relate to the smaller amount of tissue ablation and the lower temperatures used. This result should be interpreted with caution given the lack of data on sexual activity before treatment, although in randomised controlled trials the two groups should be balanced at baseline. Avoidance of these adverse effects was balanced by increases in others, particularly the need for continued catheterisation and reoperation. Laser coagulation was associated with a higher risk of urinary retention, which might reflect prostatic oedema subsequent to tissue necrosis^w13^ and this might also explain the higher rate of urinary tract infection after this procedure.^7^ The durability of symptomatic benefit after minimally invasive treatments was poor compared with transurethral resection, with rates of reoperation over 12 months of 11%, 8%, and 7% for laser coagulation, transurethral microwave treatment, and transurethral needle ablation, respectively, compared with an absolute rate of 3% for patients after transurethral resection. This represents a major disadvantage of these techniques, particularly if the rate of treatment failure continues to increase with longer follow-up, as suggested by several studies reporting outcome at two and five years after transurethral microwave treatment.^w8 w9 w18 w20 w21^ This finding provides a further argument against these technologies as alternatives to transurethral resection but might favour their use as a preliminary treatment, with transurethral resection used for treatment failures. Trials of incorporating repeated treatments with minimally invasive devices should perhaps be encouraged.

Transurethral microwave treatment, transurethral needle ablation, and laser coagulation were associated with shorter duration of operation and hospital stay, and transurethral microwave treatment can even be carried out as a day procedure, which might be advantageous to patients, society, and healthcare providers.^8^ This, together with the lower risk of serious complications, suggests that minimally invasive treatments might offer significant cost savings over transurethral resection, but the importance of these savings would need to be set against differences in effectiveness and subsequent treatment costs such as need for reoperation and safety within a formal economic evaluation. In addition, better managed pathways and improved perioperative care might mitigate against these perceived advantages of minimally invasive treatments.

### Strengths and limitations

We could not assess the clinical effectiveness of other minimally invasive treatments—such as high intensity focused ultrasound, water thermotherapy, and transurethral ethanol ablation of prostate—because of the limited data available. If these techniques seem to hold promise, methodologically sound randomised controlled trials comparing them with current practice would be necessary.

Also we could not assess the extent of publication bias as most of the available evidence (53 randomised controlled trials) was excluded because it was reported only in abstract form. Unpublished reports tend to show less positive results^9^ and so the exclusion of these abstracts might have introduced bias in favour of the newer interventions. Although 22 trials were eligible, most had suboptimal reporting or methods and recruited small numbers of patients, with many failing to report point estimates and measures of variability, hindering the calculation of a weighted mean difference. If these details were made available, effect sizes might be different. This is another reason why adherence by authors and journals to CONSORT guidelines would greatly aid the conduct of robust meta-analyses.

Heterogeneity in results for the primary outcome measure of reduction in symptom score presented problems in deriving a valid meta-analysis, which we overcame by using a random effects model. Clinical reasons for this heterogeneity include differences in baseline symptoms score between studies and failure to control for other variables that might result in greater symptomatic improvement, such as initial prostate volume and the presence of urodynamic obstruction.

Other methodological limitations resulted from variation in inclusion criteria, operative technique, and treatment protocols between trials investigating the same technology. This was particularly true for laser coagulation, for which many trials failed to report power settings, temperature, and site or duration of laser application. Such limited descriptions of technologies made it hard to determine whether they were truly minimally invasive or involved some immediate tissue removal. This is an important possible explanation for the statistical heterogeneity that was common to some of the analyses.

Though our conclusions generally concur with the other recently published reviews,^10^ ^11^ we found some differences in effect estimates but the direction of effect is generally consistent. One exception was the study by Bouza and colleagues that compared transurethral needle ablation with transurethral resection of the prostate.^10^ They reported a considerably higher risk of urinary retention after transurethral resection and a different direction of effect for urinary tract infections favouring transurethral needle ablation. These marked differences might be caused by different objectives and inclusion criteria between reviews and because Bouza and colleagues combined results from non-randomised and randomised studies to provide a single pooled estimate in the study. This type of analysis can generate biased estimates of the treatment effect and might decrease precision.^9^

### Conclusion

We used the best available techniques to identify, review, and meta-analyse available data concerning the therapeutic effect of minimally invasive surgical treatments for benign prostatic enlargement compared with transurethral resection of the prostate. Our results suggest these treatments should be used earlier in the care pathway and therefore further studies to determine advantage over drug treatment or policies that include the option of sequential treatments are needed. For the NHS, the use of these minimally invasive options is not otherwise appropriate and transurethral resection of the prostate or other endoscopic techniques that result in immediate tissue removal should be preferred.

**What is already known on this topic**

Transurethral resection of the prostate is the current standard surgical procedure for men with clinically benign prostatic enlargement
It provides a consistent high likelihood of improvement, which persists in the long term but is associated with relatively high risk of adverse events
Minimally invasive treatments have been tried in clinical practice

**What this study adds?**

Minimally invasive treatments result in poorer improvement in symptoms and urodynamic parameters than transurethral resection but cause fewer adverse events
Rates of reoperation are higher after a minimally invasive treatment than after transurethral resection

## Notes

We thank Bronwyn Davidson for secretarial support.

Contributors: TL coordinated the study, reviewed the effectiveness of the technologies, carried out data analyses, and wrote the manuscript. RP provided clinical advice and wrote the manuscript. LV and AG provided clinical or methodological advice and commented on drafts of the manuscript. CF developed and ran the search strategies and was responsible for obtaining papers and reference management. GMacL provided statistical support and advice. JN’D led and coordinated all aspects of the project. Nigel Armstrong, Ghulam Nabi, Mark Deverill, Samuel McClinton, Susan Wong, Angela Coutts, and Graham Mowatt (other members of the BPE team) contributed to development of review protocol, data abstraction, clinical content advice, and commented on analysis and interpretation of results. TL and JN’D are guarantors.

Ethical approval: Not required.

Provenance and peer review: Not commissioned; externally peer reviewed.

## Notes

**Cite this as: ***BMJ* 2008;337:a1662
